# Free Fatty acid-induced disruption of hepatic vitamin D metabolism impairs bone homeostasis in an in vitro 3D human liver–bone model

**DOI:** 10.1007/s00204-026-04431-x

**Published:** 2026-05-13

**Authors:** Mohammad Majd Hammour, Lisa Herzberger, Yuxuan Xin, Guanqiao Chen, Melike Tombaz, Sabrina Ehnert, Fabian Springer, Georg Damm, Massoud Vosough, Jan G. Hengstler, Ursula Müller-Vieira, Andreas K. Nüssler, Romina H. Aspera-Werz

**Affiliations:** 1 Department of Traumatology, Siegfried Weller Institute, BG Clinic Tübingen, Eberhard Karls University, 72076 Tübingen, Germany; 2 Boehringer Ingelheim Pharma GmbH & Co. KG, NCE, Biberach an der Riss, Germany; 3 Department of Diagnostic and Interventional Radiology, University Hospital Tübingen, Hoppe- Seyler-Str. 3, 72076 Tübingen, Germany; 4 Department of Radiology, BG-Klinik Tübingen, Eberhard Karls University, 72076 Tübingen, Germany; 5 Department of Hepatobiliary Surgery and Visceral Transplantation, Thoracic and Vascular Surgery, Clinic for Visceral, Leipzig University Medical Center, 04103 Transplant, Leipzig, Germany; 6 Department of Regenerative Medicine, Cell Science Research Center, Royan Institute for Stem Cell Biology and Technology, ACECR, Tehran, Iran; 7 Experimental Cancer Medicine, Institution for Laboratory Medicine, Karolinska Institutet, Stockholm, Sweden; 8 Leibniz Research Center for Working Environment and Human Factors (IfADo), 44139 Dortmund, Germany

**Keywords:** MASLD, Vitamin D, Liver spheroids, In vitro model, Metabolic activity, one health

## Abstract

Metabolic dysfunction-associated steatotic liver disease (MASLD) is a systemic condition associated with compromised bone integrity. Emerging evidence suggests that disturbances in hepatic vitamin D metabolism may contribute to these skeletal impairments. However, the hepatic mechanisms driving bone deterioration remain poorly defined. This study aimed to establish a human 3D in vitro model of MASLD and demonstrate that hepatic vitamin D dysregulation adversely affects bone homeostasis. Liver spheroids composed of HepaRG cells, LX-2 stellate cells, and HUVECs were stimulated with 600 µM free fatty acids (2:1 oleic: palmitic acid) to induce MASLD-like features, validated by BODIPY staining and gene expression. MASLD model induction led to downregulation of hepatic genes regulating lipid and vitamin D metabolism. ELISA confirmed significantly reduced 25-hydroxyvitamin D levels, aligning with downregulation of *CYP2R1* and *CYP27A1*. Transcriptomic profiling of human MASLD liver biopsies validated these molecular changes. To evaluate MASLD’s systemic impact on bone, THP-1-derived macrophages and SCP-1 mesenchymal stem cells were seeded onto bone scaffolds and co-cultured with spheroids. Bone scaffolds co-cultured with MASLD spheroids showed impaired mineralization and elevated expression of bone resorption markers. These findings mirror skeletal dysfunction observed in MASLD patients and suggest a mechanistic link between hepatic vitamin D dysregulation and bone pathology. This study introduces a pioneering 3D human liver-bone co-culture model that reveals MASLD-driven disruption of hepatic vitamin D metabolism as a direct contributor to bone deterioration. This 3D model develops a powerful translational platform for decoding systemic disease mechanisms and targeting the liver-bone axis therapeutically.

**Supplementary Information:**

The online version contains supplementary material available at 10.1007/s00204-026-04431-x.

## Introduction

Metabolic dysfunction-associated steatotic liver disease (MASLD), previously termed non-alcoholic fatty liver disease (NAFLD), is now the most prevalent chronic liver condition worldwide, affecting nearly 30% of the global population (Li et al. 2023; Pipitone et al. 2023). MASLD encompasses a spectrum of hepatic pathologies from benign steatosis to steatohepatitis and progressive fibrosis, and is strongly associated with systemic metabolic disorders such as obesity, insulin resistance, and type 2 diabetes (Adinolfi et al. 2025; Cobbina and Akhlaghi 2017; Targher et al. 2024; Wree et al. 2013).

Emerging evidence links MASLD to extrahepatic complications, notably impaired bone homeostasis (Monti et al. 2024). Vitamin D insufficiency is frequently observed in MASLD patients and correlates with reduced bone mineral density and increased fracture risk (Chakrabarty et al. 2024; Su et al. 2023). Hepatic conversion of cholecalciferol to 25-hydroxy-vitamin D [25(OH)D_3_], primarily mediated by Cytochrome-P450 2R1 (CYP2R1) and CYP27A1, is a key step in vitamin D metabolism (Bouillon and Bikle 2019; Ehnert et al. 2019; Wintermeyer et al. 2016). Notably, expression of these enzymes is diminished in animal models of diet-induced steatosis and insulin resistance (Zhu et al. 2021), yet the mechanistic basis and its impact on bone integrity remain poorly understood.

Among these systemic complications, hepatic osteodystrophy has gained increasing attention. Characterized by impaired bone mineralization, altered remodeling, and increased fracture risk (Ehnert et al. 2019; Hochrath et al. 2013), reflecting the complex interplay between hepatic metabolism and skeletal health. In MASLD, this condition may be driven by disrupted vitamin D metabolism, inflammatory signaling, and altered hepatic secretion of bone-modulating factors.

Animal models have provided valuable insights into MASLD pathogenesis, but often fail to predict human outcomes due to species-specific differences in metabolism (Punt et al. 2017). Primary human hepatocyte spheroids represent the current in vitro gold standard but are limited by availability and variability (Bell et al. 2016; Godoy et al. 2013; Guo et al. 2011; Strobel et al. 2021). Current MASLD models are limited by short duration, lack of multicellular complexity, and absence of systemic organ crosstalk, hindering investigation of long-term complications such as hepatic osteodystrophy (Rezvani et al. 2023; Soret et al. 2020). Our approach leverages HepaRG-based liver spheroids integrated with bone scaffolds comprising osteoblast-like SCP-1 cells and osteoclast precursors (THP-1). This co-culture system offers a reproducible, long-term platform technology that captures multicellular complexity and systemic interactions without reliance on animal models (Chen et al. 2024; Hammour et al. 2022; Lübberstedt et al. 2011; Zahmatkesh et al. 2022).

In this study, we established this platform to investigate how MASLD-induced hepatic dysfunction affects bone formation and degradation, with a particular focus on vitamin D metabolism. We hypothesized that MASLD suppresses CYP2R1/CYP27A1 expression, reduces 25(OH)D_3_ output, and consequently impairs bone mineralization and mechanical stiffness.

## Materials and methods

If not otherwise stated, all cell culture reagents and chemicals were purchased from Sigma Aldrich (Taufkirchen, Germany) or Carl Roth (Karlsruhe, Germany). Growth factors were purchased from Peprotech (Hamburg, Germany).

### Cell culture

Undifferentiated HepaRG cells (Biopredic International, Saint Grégoire, France) were cultured according to the standard protocol provided by Biopredic (Gripon et al. 2002; Hammour et al. 2022). Briefly, HepaRG cells were grown in a T75 flask for two weeks (proliferation phase) in Williams Medium (OMNI Live Science, Bremen, Germany) supplemented with 10% fetal calf serum (FCS; 10270-106, Life Technologies), 2 mM L-glutamine, 100 U/mL penicillin/streptomycin, 5 µg/mL insulin (Novo Nordisk, Bagsværd, Denmark), and 50 µM hydrocortisone (Pfizer, New York, USA).

LX-2 cells (Merck Millipore, Darmstadt, Germany) were cultured in Dulbecco’s Modified Eagle’s Medium containing 2% FCS, 2 mM L-Glutamine, and 100 U/mL Penicillin/Streptomycin.

HUVEC cells (PromoCell, Heidelberg, Germany) were cultured in a T75 flask pre-coated with 0.1% gelatin in Endothelial Cell Growth Basal Medium 2 (PromoCell). The medium was supplemented with 2% FCS, 1% Antibiotic/Antimycotic (PAA Laboratories, Toronto, Canada), 0.5 ng/mL human Vascular Endothelial Growth Factor 165, 10 ng/mL human fibroblast growth factor, 5 ng/mL human Epidermal growth factor, 20 ng/mL recombinant human insulin-like growth factor, 22.5 µg/mL Heparin (Leo Pharma, Bellerup, Denmark), 0.2 µg/mL Hydrocortisone, and 1 µg/mL L-ascorbic acid.

THP-1 monocytic cells (DSMZ, Braunschweig, Germany) were cultured in RPMI-1640 medium supplemented with 5% FCS.

SCP-1 mesenchymal stem cells, kindly provided by Prof. Dr. Matthias Schieker (Böcker et al. 2008), were maintained in MEM α medium (OMNI Life Science) supplemented with 5% FCS.

All cells were cultured at 37 °C in a humidified atmosphere with 5% CO_2_ until confluence, and the culture medium was changed three times a week.

## Generation of liver spheroids

Liver spheroids were generated as described previously (Chen et al. 2024; Zahmatkesh et al. 2022). Briefly, non-adherent agarose microwells were prepared using 2% of warm agarose. A polydimethylsiloxane mold with 300 pyramidal microwells (each 800 μm in diameter) was pressed into the agarose. After the agarose solidified, the mold was gently removed, and the plate was sterilized under UV light for 1 h.

For seeding liver spheroids, a cell suspension of HepaRG, LX-2, and HUVEC cells was prepared in a 4:2:1 ratio. The seeding density was 1000 cells per microwell. The plates were centrifuged at 1200 rpm for 3 min to settle the cells in the microwells and ensure even distribution. The cells were cultured for two days in a 50:50 mixture of HepaRG and HUVEC culture medium. For the next two weeks, 1.7% dimethyl sulfoxide (DMSO) was added to the medium to induce HepaRG cell differentiation. The medium was changed three times per week.

## MASLD induction in liver spheroids

A protocol for inducing hepatic steatosis in primary human hepatocytes was adapted for MASLD induction in liver spheroids (Nagumalli et al. 2022; Rennert et al. 2020). Briefly, after the differentiation of liver spheroids, the culture medium was replaced with control or free fatty acids (FFA)-containing liver spheroids medium (50:50 mix of HepaRG and HUVEC culture medium) for 7 days. Stimulation was repeated with every medium change, three times a week. The FFA mix consists of 0.6 mM oleic and palmitic acids at a 2:1 ratio, dissolved in Methanol at 50 °C, stored at -20 °C, and thawed once at 50 °C before use. As a control, spheroids were treated with methanol alone.

## Scaffold preparation and sterilization

Human platelet-rich plasma (hPRP) scaffolds were prepared as described previously (Chen et al. 2024; Häussling et al. 2019). Briefly, a pre-cooled mixture of 16% poly (2-hydroxyethyl methacrylate) (pHEMA), 0.3% bisacrylamide, and 0.25 g/L hPRP was prepared and incubated on ice. After 30 min, di-sodium hydrogen phosphate buffer was added to the solution to reach a final concentration of 0.3 M. Polymerization was initiated by 0.1% glutaraldehyde, 0.2% ammonium persulfate, and 0.2% Tetramethylethylendiamin. The mixture was transferred into molds, frozen at − 18 °C (≥ 12 h), deep frozen at − 80 °C for 1 h, and cut into discs (3 mm × 6 mm). The scaffolds were mineralized in 1 M CaCl_2_ for 24 h, sterilized in ethanol (70%, 12 h), and washed three times with phosphate-buffered saline (PBS).

## Bone scaffold co-culture

Sterile hPRP scaffolds were placed in 48-well plates. THP-1 monocytes (8 × 10^4^ cells in 15 µL) were seeded onto the center of the scaffold and allowed to attach for 4 hours before adding 500 µL of THP-1 medium supplemented with 200 nM of phorbol-12-myristate-13-acetate (PMA). After 24 h, SCP-1 mesenchymal stem cells (1 × 10^4^ cells in 15 µL) were seeded onto the center of the scaffold. After 4 hours attachment period, bone differentiation medium was added. This medium consisted of a 1:1 mixture of RPMI‑1640 and MEMα, supplemented with 1% FCS, 200 µM L-ascorbate-2-phosphate, 5 mM β-glycerophosphate, 25 mM HEPES, 1.5 mM CaCl_2_, and 20 ng/mL Cholecalciferol (Chen et al. 2024; Haussling et al. 2021). To ensure bone scaffold exposure to MASLD-derived factors while avoiding direct FFA effects, bone scaffolds were transferred weekly to freshly prepared MASLD-induced liver spheroids. This approach maintained continuous exposure to hepatic-secreted mediators throughout the 28-day co-culture period.

### Mitochondrial activity

Resazurin conversion was used to measure cell viability as previously described (Chen et al. 2024). The liver spheroids and the bone scaffolds were washed with PBS and incubated in 0.0025% w/v resazurin in PBS for 120 min at 37 °C. After incubation, 100 µL of supernatant was transferred to a 96-well plate, and fluorescence was measured at 544 nm excitation and 590 nm emission using an OMEGA plate reader (BMG Labtech, Ortenberg, Germany).

## DNA isolation and quantification

For cell viability assessment and normalization, DNA quantification was performed as previously described (Chen et al. 2024; Meeker et al. 2007). Liver spheroids and bone scaffolds were washed with PBS and lysed with a heated (98 °C) 50 mM NaOH solution. The samples were frozen at -20 °C. The following day, thawed samples were neutralized with 0.1 M Tris (pH 8) and centrifuged at 14,000× g for 10 min to remove impurities. DNA concentration was then quantified using an OMEGA plate reader.

## Lactate dehydrogenase

Following the manufacturer’s instructions, lactate dehydrogenase (LDH) was measured using the CytoTox-ONE Homogeneous Membrane Integrity Assay (Promega, Madison, USA). Briefly, 100 µL of cell culture medium was transferred to a 96-well plate, and an equal volume of CytoTox-ONE Reagent was added. After 15 min at room temperature (RT), 50 µL of stop solution was added. Subsequently, fluorescence was measured at 560 nm excitation and 590 nm emission using an OMEGA plate reader.

### Live-dead staining

Live-dead staining was performed using Calcein AM and ethidium bromide to assess the viability of liver spheroids (Sreekumar et al. 2018). Liver spheroids were washed three times with PBS and incubated with 2 µM Calcein AM and 4 µM ethidium bromide in PBS for 30 min at 37 °C. Images were captured using the EVOS FL fluorescence microscope (Life Technologies, Darmstadt, Germany).

### Fatty acid quantification and fluorescence microscopy

Intracellular lipid accumulation was visualized using BODIPY 493/503 (Das et al. 2023). Liver spheroids were washed three times with PBS and stained with 5 µM BODIPY and 16 µM Hoechst 33,342 in PBS for 30 min at 37 °C. Images were captured using the EVOS FL fluorescence microscope, and fluorescence was measured at an excitation wavelength of 485 nm and emission at 520 nm for BODIPY staining, while Hoechst fluorescence was measured at an excitation of 355 nm and emission at 460 nm using an OMEGA plate reader.

### Immunofluorescence characterization of liver spheroids

Liver spheroids were fixed with 4% paraformaldehyde for 30 min at room temperature, permeabilized with 0.2% Triton X-100 for 30 min, and blocked with 5% BSA in PBS for 1 h at room temperature. The spheroids were then incubated overnight at 4 °C with primary antibodies diluted 1:100 in PBS against albumin (Santa Cruz, Heidelberg, Germany; sc-50535), CD31 (Santa Cruz, sc-376764), and vimentin (Cell Signaling, Frankfurt, Germany; 5741) overnight at 4 °C. After washing, samples were incubated with the corresponding Alexa Fluor-conjugated secondary antibodies (Invitrogen, Darmstadt, Germany; A21206 & A10667) diluted 1:1000 in PBS. F-actin was visualized using phalloidin, and nuclei were counterstained with Hoechst 33,342. Images were acquired using Logos Biosystems microscope (CELENA X, Villeneuve d’Ascq, France) under identical settings for control and MASLD spheroids.

### mRNA expression analysis

*QuantiGene Plex (QGP) Assay*. mRNA expression was assessed using the QuantiGene Plex assay (Thermo Fisher Scientific, Karlsruhe, Germany) according to the manufacturer’s instructions. Cells were washed twice with ice‑cold PBS and lysed in Cell Extraction Buffer (Invitrogen, Darmstadt, Germany) containing Halt Protease Inhibitor (Thermo Scientific) for 10 min on ice. Lysates were mixed with diluted Lysis Working Mixture (2:1) and incubated (50 °C, 30 min), then hybridized overnight (54 °C, 240 rpm) with target‑specific magnetic capture beads. Following magnetic separation and washing, Pre-Amplifier, Amplifier, and Label Probe solutions were applied sequentially (each for 1 h at 50 °C and 240 rpm), and the signal was developed with SAPE Working Reagent (30 min at RT, 600 rpm). Afterward, the signals were measured by the Luminex™ 200™ Instrument. For QuantiGene Plex analysis, target signals were normalized to the internal housekeeping genes *RPL32*, *GUSB*, and *EIF4E2.* Expression level of target genes are presented as normalized relative expression values.

*RT-PCR*. Total RNA was extracted from liver spheroids using self-made Trifast reagent (Rodriguez-Ezpeleta et al. 2009). RNA quantity and purity were assessed using an OMEGA plate reader. Subsequently, 2.5 µg of purified RNA was used for cDNA synthesis using a commercially available cDNA reverse-transcription kit (Thermo Fisher Scientific) according to the manufacturer’s instructions. PCR was performed with primer sets listed in Table 1 and using the Red HS Taq Master Mix (Biozym, Vienna, Austria) following the manufacturer’s protocol. The amplified PCR products were visualized on an agarose gel containing ethidium bromide. Densitometric analysis of the bands was performed using ImageJ software (V. 1.54f, National Institutes of Health, USA) for relative quantification of gene expression. The data were normalized to the expression of the housekeeping gene *HPRT1* and expressed as fold change relative to the corresponding control group.

**Table 1:** Primer sequences used for the reverse transcriptase PCR

Gene	Forward/ Reverse sequences	GenBank accession	Tm^[a]^ (°C)	Cycles
*HPRT1*	CCTGGCGTCGTGATTAGTGA CGAGCAAGACGTTCAGTCCT	NM_000194.2	58	30
*CYP27A1*	GTGGACACGACATCCAACAC ATGATCCGGGAGTTTGTGG	NM_000784.4	63	35
*CYP2R1*	TGGAAGCTTTGGAGAGCTGA ATCTCTCCGTACACCTGGCT	NM_024514.5	64	35

^[a]^Annealing temperature

### Dot blot analysis

Secreted protein levels in the cell culture supernatants were quantified using dot blot analysis as previously described (Chen et al. 2024). Briefly, 100 µL of each sample was spotted onto a nitrocellulose membrane using a 96-well dot-blotter (Carl Roth, Karlsruhe, Germany). The transfer of proteins was confirmed by Ponceau staining. The membrane was blocked with 5% bovine serum albumin in TRIS-buffered saline/Tween 20 (TBS-T) for 1 h, then incubated with primary antibodies against tissue nonspecific alkaline phosphatase (AP; sc 23430; Santa Cruz Biotech, Heidelberg, Germany), Procollagen type 1 n-terminals propeptide (PINP; abx131414; Abbexa, Cambridge, UK), or Tartrate-resistant acid phosphatase (TRAP; sc-376875, Santa Cruz Biotechnology, Heidelberg, Germany) (1:1000 in TBS-T) for 1 h at room temperature and overnight at 4 °C. After washing, the membrane was incubated with the corresponding HRP-conjugated secondary antibodies (1:10000 in TBS-T) for 1 h at room temperature. Protein dots were visualized using enhanced chemiluminescence reagent and imaged with a CCD camera (INTAS, Göttingen, Germany). Dot intensities were quantified using ImageJ software, with values normalized to the total proteins quantified using Ponceau staining for each sample.

### Vitamin D metabolism activity using enzyme-linked immunosorbent assays

Liver spheroids were washed three times with PBS and incubated with 20 µM vitamin D in plain Williams medium. After 4 h, the supernatants were stored at -80 °C for analysis, and the spheroids were collected for DNA isolation. An enzyme-linked immunosorbent assay (ELISA) kit for 25(OH)D3 (Immunodiagnostic Systems, Frankfurt, Germany) was used according to the manufacturer’s protocol. Absorbance was measured at 450 nm with an OMEGA Plate Reader, and concentrations were calculated from standard curves generated with known concentrations of 25(OH)D3. The concentrations were normalized to the total DNA of each sample condition.

### Cytokine quantification by ELISA

Supernatants from control and FFAs-treated liver spheroids were collected after 7 days of exposure and stored at − 80 °C until analysis. Secreted Interleukin 6 (IL-6), Tumor necrosis factor α (TNF-α), Interleukin 1β (IL-1β), and Interferon-γ (IFN-γ) levels were quantified using commercially available ELISA kits according to the manufacturers’ instructions (IL-6, BioLegend, Koblenz, Germany, CAT# 430504; TNF-α, BioLegend, CAT# 430204; IL-1β, BioLegend, CAT# 437003; IFN-γ, BioLegend, CAT# 430104). Absorbance was measured using an Omega Plate Reader, and cytokine concentrations were calculated from standard curves generated with the respective standards.

### Bioinformatics RNA-sequencing analysis

RNA-Seq analysis was performed on liver biopsies to identify genes affected by MASLD. Raw sequence reads were downloaded from the NCBI (accession number PRJEB58091) (European Bioinformatics 2023). The reads were aligned to the human reference genome (GRCh38) using “HISAT2” (version 2.1) (Kim et al. 2019). Gene expression levels were quantified by counting mapped reads using “featureCounts” (version 2.0.1) from the Subread package (Liao et al. 2014). Raw counts were normalized to Transcripts Per Million (TPM) for between-sample comparisons. Differential expression analysis was conducted using “DESeq2” (version 1.30.1) (Love et al. 2014) in R (version 4.0.3).

### Alkaline phosphatase (AP) activity

AP activity was measured based on the conversion of 4-nitrophenyl phosphate to 4-nitrophenol (Aspera-Werz et al. 2018). Scaffolds were washed with PBS and incubated with AP buffer (3.5 mM 4-nitrophenyl phosphate, 50 mM glycine, 1 mM MgCl_2_, 100 mM TRIS, pH 10.5) at 37 °C for 2 h. Absorbance was measured at 405 nm with an OMEGA Plate Reader. Values normalized to DNA content.

### Carbonic anhydrase II (CAII) activity

CAII activity was assessed by incubating washed scaffolds in CAII buffer (12.5 mM TRIS, 75 mM NaCl, 200 mM 4-nitrophenyl acetate; pH 7.5) at 37 °C for 15 min (Zhu et al. 2020). Absorbance was measured at 405 nm with an OMEGA Plate Reader. Data were normalized to DNA content.

### CT analysis of mineral content

Mineral content was analyzed using a 128-slice clinical CT scanner (SOMATOM Definition Edge, Siemens). Scanning was performed at 80 kV, 500 mAs, 16 × 0.3 mm acquisition, and 0.4 mm slice thickness. Images were reconstructed with SAFIRE (Grade 5, V80u kernel). DICOM files were processed in ImageJ. Integrated density values of each scaffold were determined and normalized against a phantom reference block (EFP-06-96) (Chen et al. 2024).

### Mechanical testing

Scaffold stiffness was determined by compression testing using a ZwickiLine Z2.5TN (Zwick GmbH, Ulm, Germany). Scaffolds were compressed uniaxially at 5 mm/min to 10% of original height. Force-displacement data were recorded in real time. Young’s modulus (MPa) was calculated as previously described (Chen et al. 2024):

$${\mathrm{Youngn's}}\:{\mathrm{modulus}}\:\left( {MPa} \right) = \frac{{{\mathrm{Applied}}\:{\mathrm{Force}}\left( N \right) \times {\mathrm{Initial}}\:{\mathrm{Height}}\left({\mathrm{mm}} \right)}}{{{\mathrm{Scaffold}}\:{\mathrm{Area}}\:({\mathrm{mm}}^{2} ) \times {\mathrm{Change}}\:{\mathrm{in}}\:{\mathrm{Height}}\left( {\mathrm{mm}} \right)}}$$

### Statistical analysis

All values are represented as Tukey’s boxplots of three independent experiments (*N* = 3, *n* = 3). Statistical significance was tested using the Kruskal-Wallis test followed by Dunn’s multiple comparison test, or by the Mann-Whitney test where appropriate (GraphPad Prism 8 Software, San Diego, USA). All statistical comparisons were performed two-sided in the context of exploratory data analysis, using *p* < 0.05 (*), *p* < 0.01 (**), and *p* < 0.001 (***) as levels of significance.

## Results

### Effects of oleic and palmitic acid treatment on liver spheroids viability

**Fig. 1:**
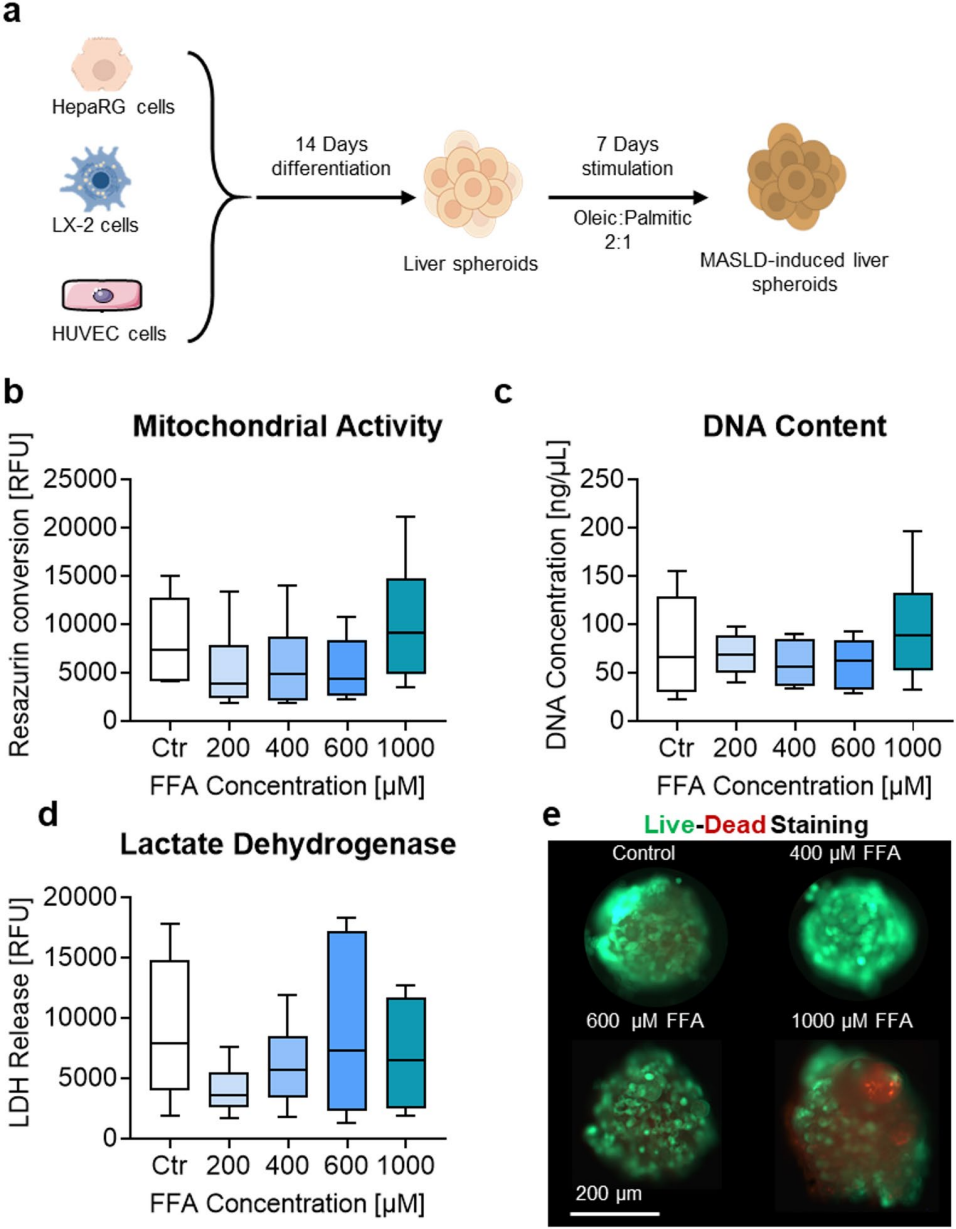
Viability assessment of liver spheroids after 7 days of oleic: palmitic acid treatment. Liver spheroids were treated for 7 days with a 2:1 oleic: palmitic acid mixture at concentrations of 0 µM (control, Ctr.) to 1000 µM. **a** Schematic overview of the experimental design. **b** Mitochondrial activity measured by resazurin conversion. **c** DNA content quantification. **d** Cell membrane integrity is assessed by lactate dehydrogenase (LDH) release. **e** Live-Dead staining using calcein‑AM (green, viable cells) and ethidium bromide (red, dead cells). Scale bar: 200 μm. Data are presented as Tukey boxplots (*N* = 3 independent experiments, *n* = 2 technical replicates). Statistical significance was determined using the Kruskal-Wallis test

To establish a physiologically relevant MASLD model, 3D liver spheroids were generated from HepaRG cells, LX‑2 stellate cells, and HUVEC endothelial cells in a 4:2:1 ratio. To determine a non-cytotoxic yet metabolically active concentration of free fatty acids (FFAs), the 14-day differentiated spheroids were treated for 7 days with a 2:1 oleic to palmitic acid mixture (0-1000 µM). The experimental design for MASLD induction is shown in Fig. 1a. Quantitative viability assays revealed no significant decrease in resazurin conversion (Fig. 1b), DNA content (Fig. 1c), or lactate dehydrogenase (LDH) release (Fig. 1d) at concentrations used for the induction compared to vehicle controls (*p* > 0.05). Live-Dead staining (Fig. 1e) showed predominant esterase activity (Calcein AM green staining for live cells) and minimal free DNA (Ethidium bromide red staining for dead cells) at FFAs concentrations ≤ 600 µM. In contrast, 1000 µM FFAs induced a marked ethidium bromide signal, indicating membrane compromise and cell death. Based on these findings, 1000 µM was excluded from further MASLD induction experiments. To assess whether FFAs-induced steatosis was reversible, liver spheroids were exposed to 600 µM FFAs for 7 days and then cultured in Medium without FFAs. BODIPY quantification showed that intracellular lipid accumulation decreased significantly after withdrawal of FFAs (Supplementary Fig. S1), indicating that the steatotic phenotype is physiologically reversible.

#### Induction of steatosis and altered hepatic lipid metabolism in liver spheroids

**Fig. 2:**
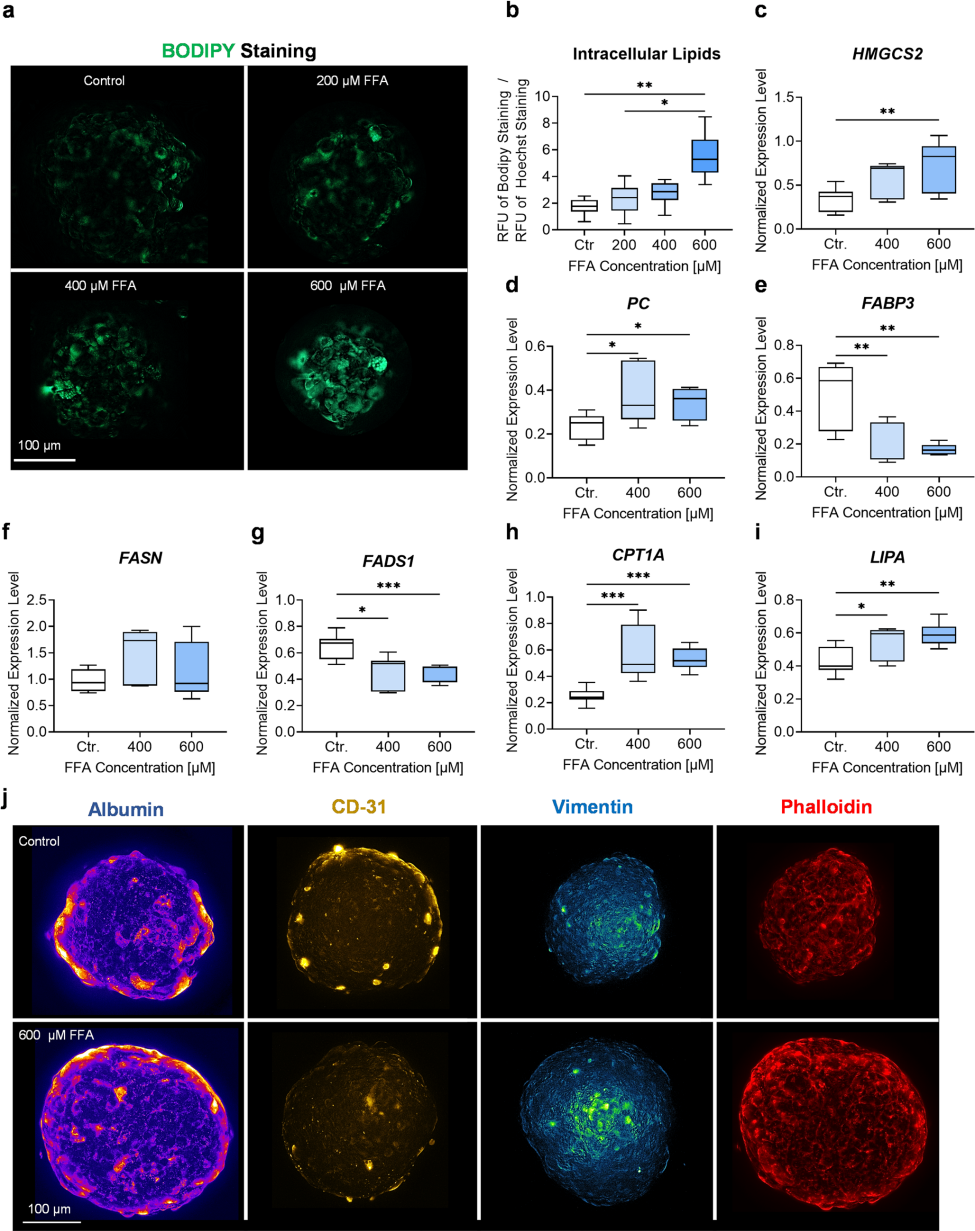
FFA‑induced steatosis and changes in lipid metabolism gene expression in liver spheroids. Liver spheroids were treated for 7 days with a 2:1 oleic: palmitic acid mixture at concentrations of 0 µM (control, Ctr.) to 600 µM. **a** BODIPY 493/503 staining visualizing intracellular neutral lipid accumulation. Scale bar: 100 μm **b** Quantification of BODIPY fluorescence intensity. **c–i** Relative mRNA expression levels determined by QuantiGene Plex assay and normalized to the internal housekeeping genes *RPL32*, *GUSB*, and *EIF4E2*. **c** 3‑hydroxy‑3‑methylglutaryl‑CoA synthase 2 (*HMGCS2*). **d** Pyruvate carboxylase (*PC*). **e** Fatty acid binding protein 3 (*FABP3*). **f** Fatty acid synthase (*FASN*). **g** Fatty acid desaturase 1 (*FADS1*). **h** Carnitine palmitoyltransferase 1 A (*CPT1A*). **i** Lipase A, lysosomal acid type (*LIPA*). **j** Representative immunofluorescence images of control and 600 µM FFAs-treated liver spheroids stained for albumin (pseudo colored in ImageJ with Magenta hot), CD31 (pseudo colored in ImageJ with Yellow hot), vimentin (pseudo colored in ImageJ with Green fire Blue), and phalloidin (pseudo colored in ImageJ with Red hot). Scale bar: 100 μm. Data are presented as Tukey boxplots (*N* = 3 independent experiments, *n* = 3 technical replicates). Statistical significance was determined using the Kruskal-Wallis test, with significance indicated by *p < 0.05, **p < 0.01, ***p < 0.001

The exposure of liver spheroids to FFAs for 7 days resulted in a clear, dose‑dependent accumulation of intracellular neutral lipids. BODIPY 493/503 staining showed markedly increased fluorescence intensity at 400 µM and 600 µM compared to the control (Fig. 2a). Quantitative analysis confirmed significant lipid accumulation with 600 µM FFAs (Fig. 2b). Since 200 µM FFAs exposure caused minimal lipid accumulation, this concentration was excluded from subsequent experiments.

To characterize metabolic changes associated with steatosis, we analyzed genes involved in lipid metabolism and MASLD development. Notably, treatment with FFAs significantly upregulated 3-hydroxy-3-methylglutaryl-CoA synthase 2 (*HMGCS2*) and pyruvate carboxylase (*PC*), which are involved in ketogenesis and gluconeogenesis, respectively (Fig. 2c, d). In contrast, fatty acid binding protein 3 (*FABP3*) and fatty acid desaturase 1 (*FADS1*), which are involved in fatty acid transport and desaturation, were significantly downregulated (Fig. 2e, g). Additional alterations included upregulation of fatty acid synthase (*FASN*) (Fig. 2f), modulation of carnitine palmitoyltransferase 1 A (*CPT1A*) (Fig. 2h), and increased lipase A, lysosomal acid type (*LIPA*) expression (Fig. 2i). Together, these morphological and transcriptional changes confirm that the chosen FFAs exposure effectively models the steatotic phenotype of early‑stage MASLD in a human 3D spheroid system, providing a physiologically relevant platform for mechanistic studies. Immunofluorescence staining further confirmed the multicellular composition and structural organization of the liver spheroids (Fig. 2j). Albumin staining was detected throughout the spheroids, confirming the presence of hepatocyte-like cells (Peng et al. 2025), whereas CD31-positive cells were localized to discrete regions consistent with endothelial compartmentalization (Lertkiatmongkol et al. 2016). Vimentin staining identified the stromal-like LX-2 cell population (Xu et al. 2005), and phalloidin highlighted the overall cytoskeletal organization of the spheroids (Chazotte 2010). Compared with controls, MASLD spheroids appeared larger and showed a stronger and more widespread vimentin-positive signal, suggesting remodeling of the stromal/fibrotic compartment while preserving hepatocyte and endothelial identity.

#### Altered expression of hepatic metabolism and transporter genes in MASLD liver spheroids

**Fig. 3:**
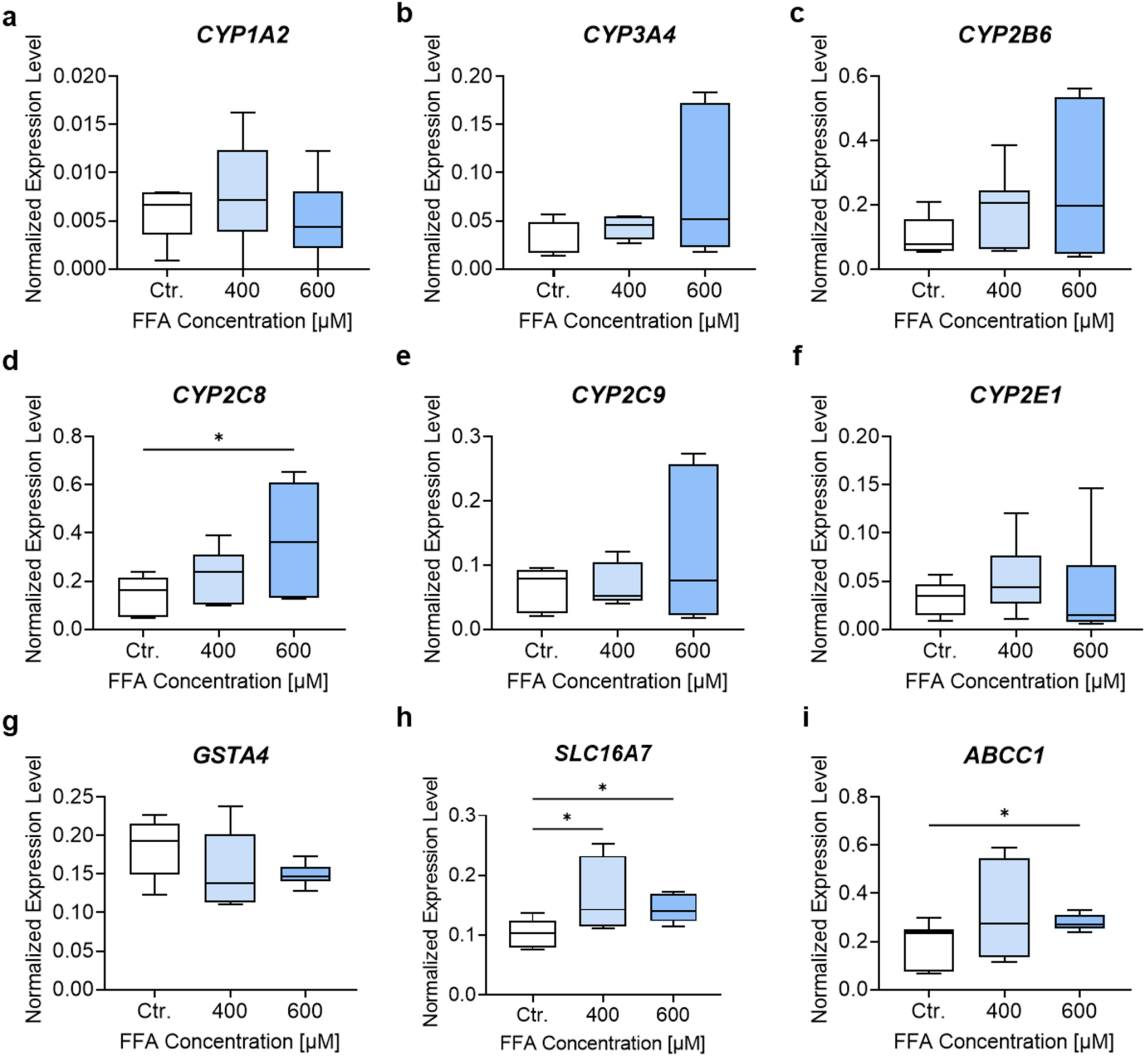
Effects of FFAs treatment on hepatic metabolism and transporter gene expression in liver spheroids. Liver spheroids were treated for 7 days with a 2:1 oleic: palmitic acid mixture at concentrations of 0 µM (control, Ctr.), 400 µM, and 600 µM. (A–I) Relative mRNA expression levels determined by QuantiGene Plex assay and normalized to the internal housekeeping genes *RPL32*, *GUSB*, and *EIF4E2*. **a** *CYP1A2*, **b** *CYP3A4*, **c** *CYP2B6*, **d** *CYP2C8*, **e** *CYP2C9*, **f** *CYP2E1*, **g** Glutathione S-transferase A4 (*GSTA4*), **h** solute carrier family 16 member 7 (*SLC16A7*), and **i** ATP-binding cassette subfamily C member 1 (*ABCC1*). Data are presented as Tukey boxplots (*N* = 3 independent experiments, *n* = 3 technical replicates). Statistical significance was determined using the Kruskal-Wallis test, with significance indicated by *p < 0.05

To evaluate the effects of MASLD induction on hepatic metabolic gene expression, we quantified mRNA levels of key phase I cytochrome P450 (CYP) enzymes, phase II detoxification enzymes, and transporters in liver spheroids following FFAs exposure (Fig. 3a-i). *CYP2C8* was significantly upregulated at 600 µM FFAs (Fig. 3d). Also, the expression of solute carrier family 16 member 7 (*SLC16A7*) and ATP-binding cassette subfamily C member 1 (*ABCC1*) was significantly increased. (Fig. 3h, i). *CYP3A4*, *CYP2B6*, and *CYP2C9* showed an increasing expression trend, but these changes did not reach statistical significance (Fig. 3b, c, e). No significant differences were observed for *CYP1A2*, *CYP2E1*, or Glutathione S-transferase A4 (*GSTA4*) under any condition (Fig. 3a, f, g). These findings indicate that steatotic injury in liver spheroids selectively induces the transcription of certain phase I enzymes and transporter genes, reflecting an adaptive yet gene-specific hepatic response to FFAs exposure.

### Increased inflammation and liver damage in MASLD liver spheroids

**Fig. 4:**
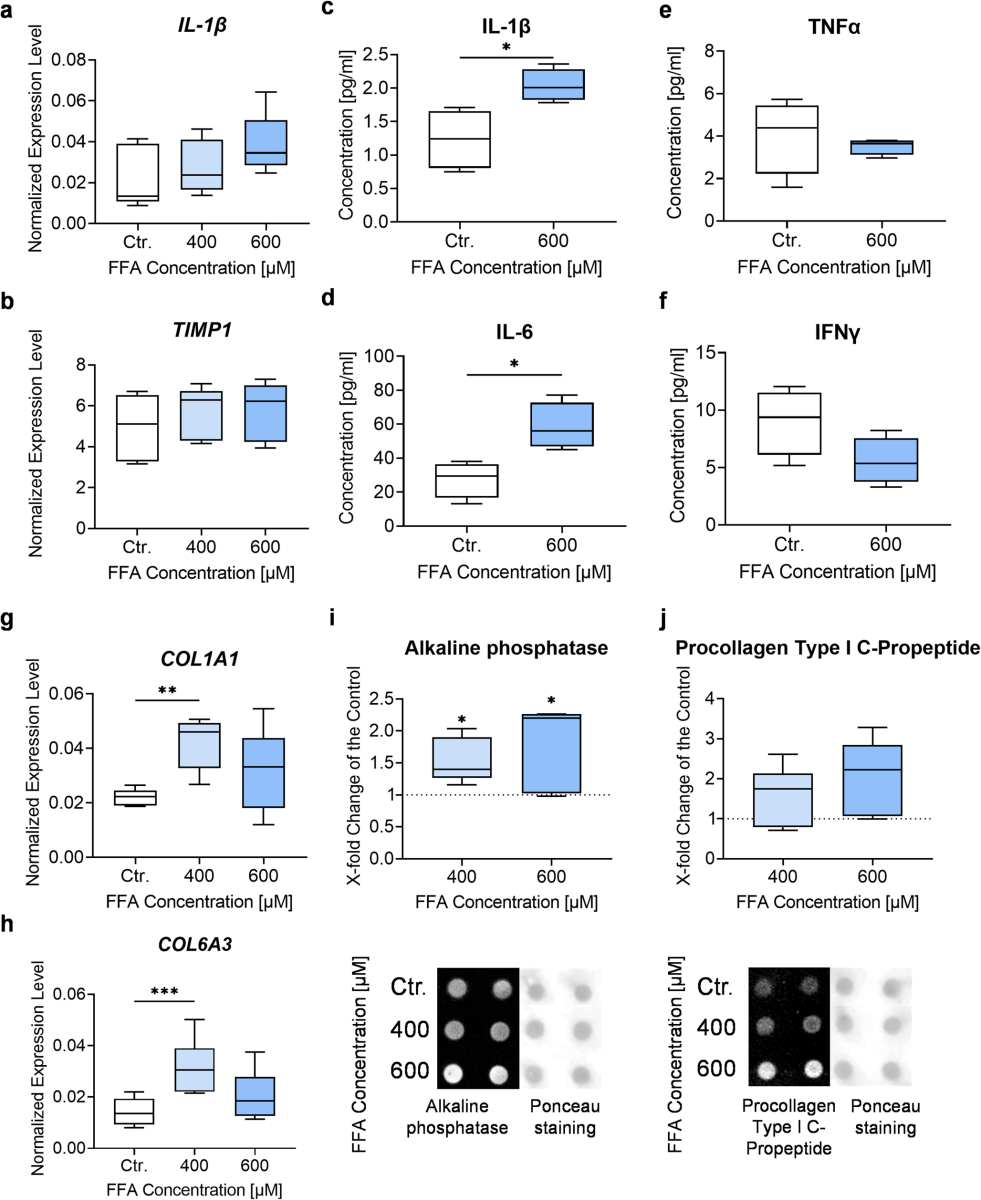
Effects of FFAs treatment on fibrosis, inflammation, and matrix remodeling markers in liver spheroids. Liver spheroids were treated for 7 days with a 2:1 oleic: palmitic acid mixture at concentrations of 0 µM (control, Ctr.), 400 µM, and 600 µM. **a**,** b**,** g**,** h** Relative mRNA expression levels determined by QuantiGene Plex assay and normalized to the internal housekeeping genes *RPL32*, *GUSB*, and *EIF4E2*. **a** interleukin 1 beta (*IL-1β*), **b** tissue inhibitor of metalloproteinases 1 (*TIMP1*), **e** collagen type I alpha 1 chain (*COL1A1*), and **f** collagen type VI alpha 3 chain (*COL6A3*). **c**–**f** Secreted cytokine concentrations determined by ELISA: **c** IL-1β, **d** interleukin 6 (IL-6), **e** Tumor necrosis factor α (TNF-α), and **f** Interferon-γ (IFN-γ). **i**,** j** Levels of secreted proteins determined by dot blot **i** Alkaline phosphatase (AP) and the representative Dot blot and Ponceau staining. **j** Procollagen type I C-propeptide (PINP) and the representative dot plot and Ponceau staining. The dashed line indicates the control. Data are presented as Tukey boxplots (*N* = 3 independent experiments, *n* = 3 technical replicates). Statistical significance was determined using the Kruskal-Wallis test or Mann-Whitney test, with significance indicated by *p < 0.05, **p < 0.01, and ***p < 0.001

To characterize liver injury and inflammatory activation during MASLD progression, we measured the expression of key pro-inflammatory and matrix remodeling genes, as well as secreted liver damage markers, in liver spheroids following FFAs exposure (Fig. 4a–j).

FFAs treatment led to a tendency toward higher mRNA levels of the inflammatory marker *IL-1β* and the tissue inhibitor of metalloproteinases (*TIMP1*); however, these differences did not reach statistical significance (Fig. 4a, b). To further assess inflammatory activation at the protein level, cytokine concentrations were quantified in spheroid supernatants. IL-6 and IL-1β levels were significantly increased under MASLD conditions compared with controls (Fig. 4c, d), confirming activation of a pro-inflammatory secretory response. In contrast, TNF-α and IFN-γ did not show significant changes (Fig. 4e, f). Together, these findings demonstrate that FFAs treatment induces not only early fibrogenic remodeling but also a measurable inflammatory response at the protein level in the liver spheroids.

In contrast, expression of fibrosis-associated markers was significantly increased: collagen type I alpha 1 chain (*COL1A1*) was upregulated at 400 µM FFAs, and collagen type VI alpha 3 chain (*COL6A3*) was elevated at both 400 µM and 600 µM FFAs (Fig. 4g, h), indicating an early fibrogenic response.

Secreted biomarker analysis revealed that alkaline phosphatase (AP) concentration was significantly increased at both 400 µM and 600 µM FFAs (Fig. 4i). Procollagen type I C-propeptide (PINP), a marker of type I collagen synthesis and extracellular matrix remodeling, also showed a trend toward increased levels in FFAs-treated spheroids **(**Fig. 4j).

The significant upregulation of major collagen genes and increased AP release, along with a trend toward increased inflammatory signals, indicate that MASLD induction in liver spheroids triggers early molecular markers of hepatocellular injury and matrix remodeling.

### Suppression of the hepatic vitamin d metabolism pathway in MASLD liver spheroids

**Fig. 5:**
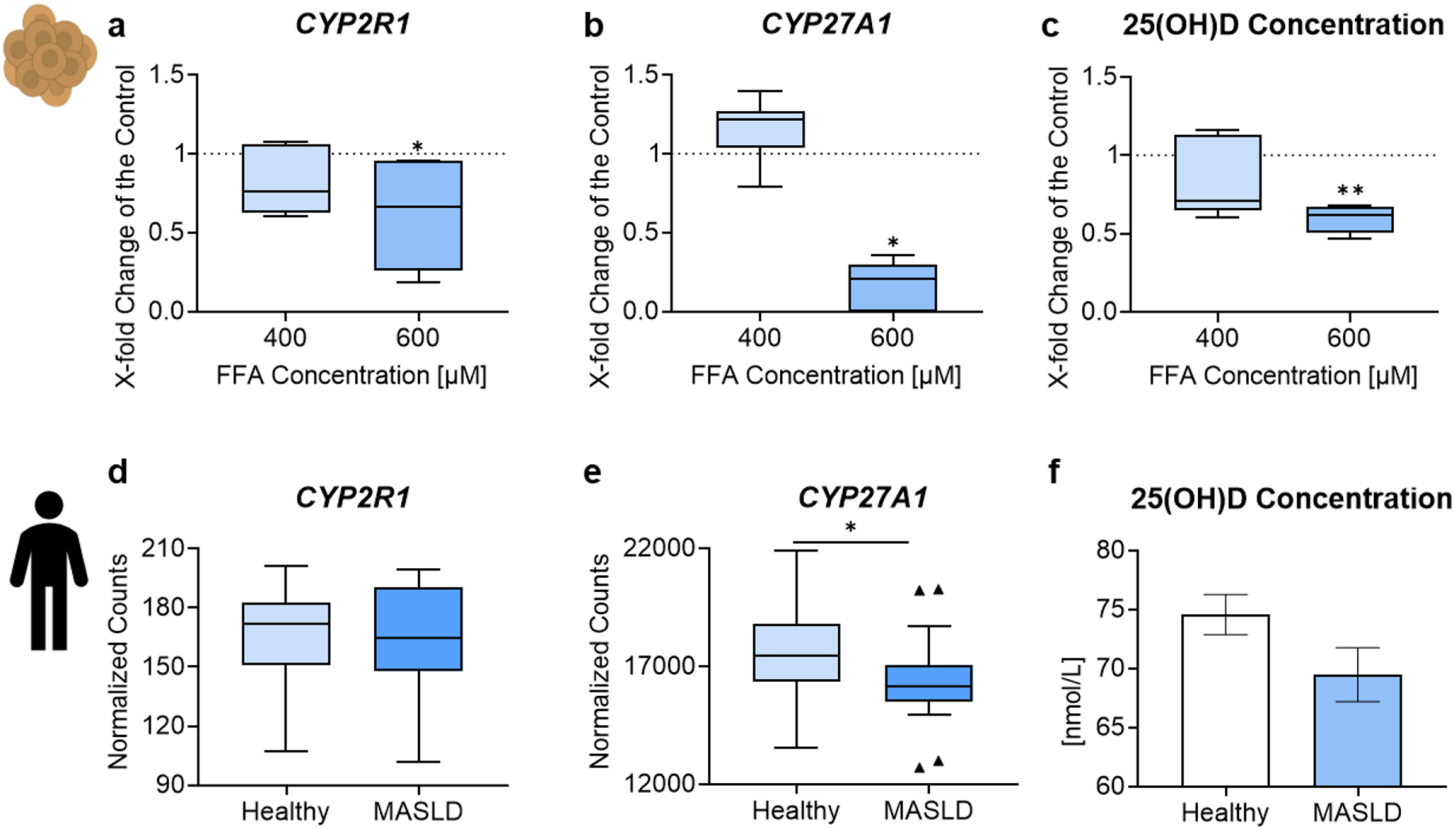
Suppression of hepatic vitamin D metabolism in MASLD: evidence from liver spheroids and human data.**a**,** b** Semi-quantitative RT-PCR band intensities normalized to HPRT1 and expressed as fold change relative to the control group in liver spheroids following FFAs treatment at 400 µM and 600 µM (*N* = 3, *n* = 3). *a* *CYP27A1. ***b** *CYP2R1.***c** X-fold change in the concentration of 25-hydroxyvitamin D [25(OH)D_3_] by liver spheroids after FFAs exposure compared to the control. **d**–**e** Human liver RNA-seq data from 7 healthy donors and 8 MASLD patients (accession number PRJEB58091). **d** *CYP2R1, ***e** *CYP27A1*. The dashed line indicates the control. Data are presented as Tukey boxplots. **f** 25(OH)D_3_ human serum concentration, data adapted from Chai et al. 2024 (Chai and Tao 2024), each bar represents the mean ± SD. Statistical significance was determined by using the Kruskal-Wallis test, with significance indicated by *p < 0.05, **p < 0.01

To determine whether MASLD affects hepatic vitamin D metabolism, we first quantified the expression of key 25-hydroxylases in liver spheroids exposed to FFAs. Both *CYP2R1* and *CYP27A1* were significantly downregulated in FFAs-treated liver spheroids compared to controls (Fig. 5a, b). Consistent with reduced enzyme expression, the metabolism of vitamin D to 25(OH)D_3_ was markedly decreased following FFAs treatment (Fig. 5c).

To assess the translational relevance of these findings, we analyzed publicly available human hepatic RNA-seq data from individuals with MASLD and matched controls, obtained from the NCBI (accession number PRJEB58091) and deposited by the European Bioinformatics Institute. Consistent with the liver spheroids results, hepatic *CYP2R1* expression remained unchanged in MASLD patients, while *CYP27A1* was significantly decreased (Fig. 5d, e). To further support the translational relevance of our findings, we included serum 25(OH)D_3_ data from MASLD patients and healthy controls, adapted from Chai et al. 2024 (Chai and Tao 2024). These data demonstrate a significant reduction in circulating 25(OH)D_3_ levels in MASLD (Fig. 5f).

Overall, these data show that MASLD causes consistent suppression of hepatic vitamin D metabolism in our liver spheroid model and is associated with reduced *CYP27A1* expression and lower 25(OH)D3 levels across the in vitro and human datasets.

## Impaired mineral density in bone scaffolds under MASLD conditions

**Fig. 6:**
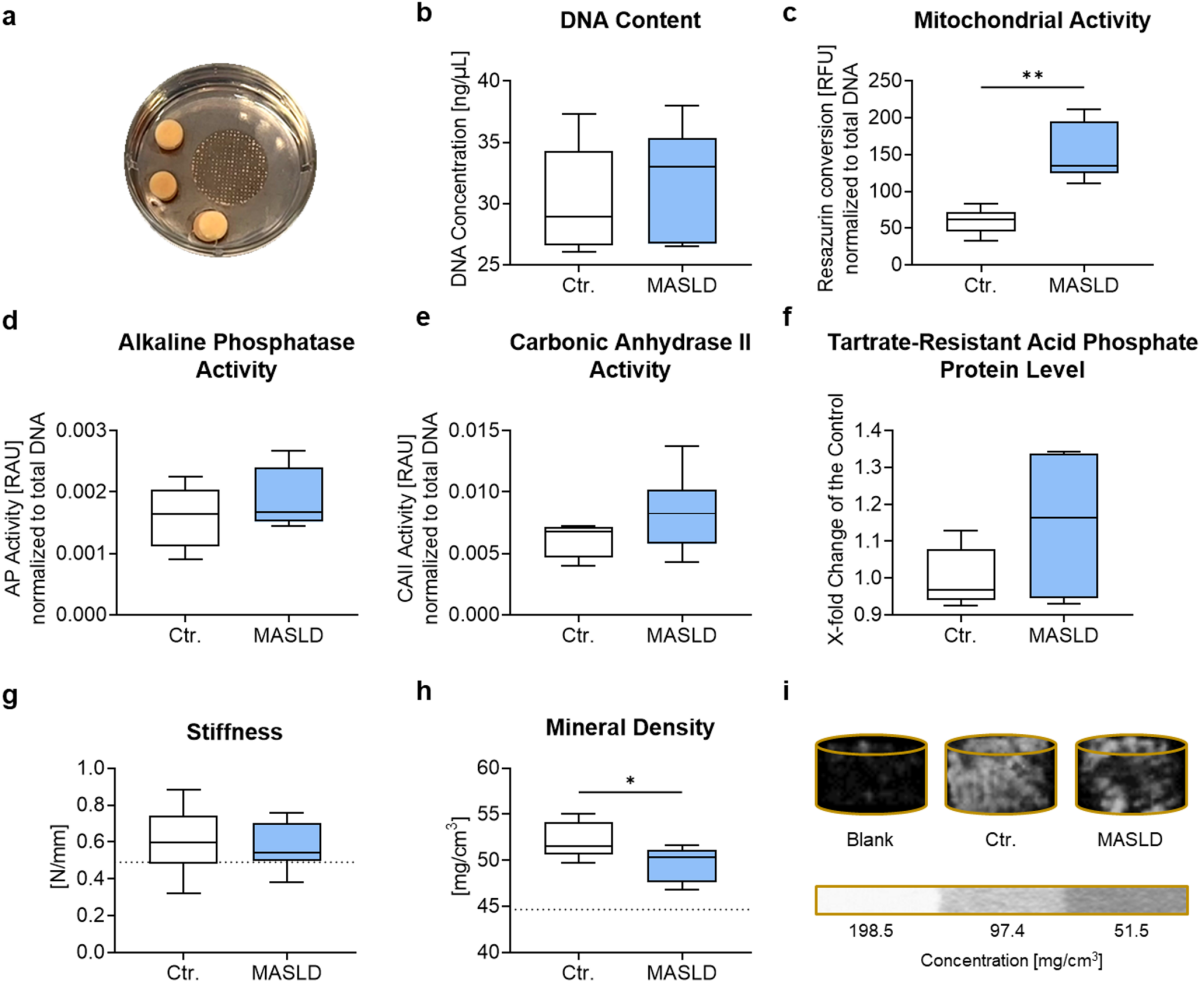
Impact of MASLD-conditioned medium on bone scaffold mineral density and metabolic function.**a** Representative image of liver spheroids and bone scaffold co-culture. **b** DNA content measured in bone scaffolds at day 21. **c** Mitochondrial activity assessed by resazurin conversion normalized to total DNA at day 21. **d** Alkaline phosphatase (AP) activity normalized to DNA at day 21. **e** Carbonic anhydrase II (CAII) activity normalized to DNA at day 21. **f** Tartrate-resistant acid phosphatase (TRAP) protein level at day 21. **g** Mechanical stiffness of bone scaffolds at day 28. **h** Mineral density of bone scaffolds measured by computed tomography at day 28. **i** Representative CT images of blank scaffold, control, and MASLD-conditioned bone scaffolds; scale indicates mineral density (mg/cm^3^). The dashed line indicates the blank scaffold. Data are presented as Tukey boxplots (*N* = 3 independent experiments, *n* = 2 technical replicates). Statistical significance was determined by using the Kruskal-Wallis test, with significance indicated by *p < 0.05, and **p < 0.01

To investigate the downstream effects of MASLD on bone, we first induced MASLD in liver spheroids and subsequently co-cultured them with bone scaffolds for up to 28 days. A representative image of the co-culture system is shown in Fig. 6a.

Following the co-culture, cellular cytotoxicity and stress were first assessed to ensure system integrity. DNA content analysis revealed no significant differences between MASLD-conditioned and control bone scaffolds (Fig. 6b), indicating comparable cell numbers and the absence of cytotoxic effects. However, mitochondrial activity was significantly elevated in MASLD-exposed scaffolds, demonstrating elevated cellular stress, as evidenced by enhanced resazurin conversion (Fig. 6c).

Assessment of early osteogenic and osteoclastic markers revealed that AP activity (Fig. 6d) showed no significant differences between MASLD-conditioned and control scaffolds. The early osteoclastic marker, carbonic anhydrase II (CAII) activity (Fig. 6e), showed a non-significant trend toward an increase in MASLD- conditioned scaffolds. Similarly, the protein level of tartrate-resistant acid phosphatase (TRAP), a late osteoclastic marker, also tended to be elevated without reaching statistical significance (Fig. 6f).

Mechanical testing of bone scaffolds indicated no significant changes in stiffness between MASLD and control conditions (Fig. 6g). In contrast, computed tomography (CT) revealed a significant decrease in mineral density in MASLD-conditioned scaffolds compared to controls (Fig. 6h). Representative CT imaging further confirmed reduced mineral accumulation in the MASLD group (Fig. 6i).

## Discussion

MASLD is increasingly recognized as a multisystemic condition, with effects that extend beyond hepatic steatosis (Monti et al. 2024). Recent research has linked MASLD to elevated risk of osteoporosis and reduced bone mineral density (Chakrabarty et al. 2024; Su et al. 2023). Yet the mechanistic link between fatty liver and impaired bone quality remains unclear. Vitamin D deficiency is a well-documented comorbidity in patients with MASLD (Barchetta et al. 2020; Lee et al. 2024). Epidemiological studies have consistently reported inverse correlations between serum 25(OH)D_3_ levels and MASLD severity (Küçükazman et al. 2014; Park et al. 2017). This suggests that hepatic metabolism, rather than solely systemic or environmental factors, contributes to vitamin D insufficiency. However, association alone has not clarified whether insufficiency is due to decreased hepatic activation, increased catabolism, or altered binding and distribution.

In this study, we developed a human 3D in vitro MASLD-bone co-culture model and identified impaired hepatic vitamin D 25-hydroxylation as a mechanistic link between MASLD and decreased bone mineralization. FFA-induced steatotic-like condition in human liver spheroids downregulated *CYP2R1* and *CYP27A1* gene expressions, decreased 25(OH)D_3_ level, and was accompanied by reduced bone scaffold mineral density. This effect is consistent with clinical associations between MASLD, vitamin D insufficiency, and the risk of osteoporosis (Lee et al. 2024; Su et al. 2023). Together with complementary analyses of human liver RNA-seq (reduced expression of *CYP27A1*), these data suggest that impaired hepatic vitamin D metabolism is a key factor in liver-bone crosstalk in metabolic disease. Importantly, the hepatic phenotype in our model aligns with in vivo observations in diet-induced steatosis, where Western diet models suppress hepatic *Cyp2r1*, leading to lower circulating 25(OH)D_3_ levels (Elkhwanky et al. 2020; Roizen et al. 2019; Zhu et al. 2021).

Our liver model tolerated FFAs concentrations up to 600 µM without significant cytotoxicity. This aligns with reported data in PHHs, which maintain viability at comparable doses (Gómez-Lechón et al. 2007; Rennert et al. 2020). In contrast, HepG2 cells exhibit marked cytotoxicity at substantially lower concentrations (Brecklinghaus et al. 2022), underscoring the superior metabolic competence of HepaRG-derived hepatocytes. HepaRG cells preserve key hepatocyte functions, including mitochondrial β-oxidation and lipid handling pathways, which likely mitigate lipotoxic stress. Furthermore, the stellate cells’ capacity for lipid storage and retinoid metabolism (Shmarakov et al. 2019) suggests a contributory role in reducing FFA-induced toxicity within the spheroids microenvironment. This multicellular interplay may more accurately recapitulate the hepatic adaptation to lipid overload observed in vivo, where stellate cells and hepatocytes cooperate to maintain lipid homeostasis.

Vitamin D pathway provides several potential points for disturbance in metabolic liver disease. While CYP2R1 and CYP27A1 mediate hepatic 25-hydroxylation (Ehnert et al. 2019; Nussler et al. 2014; Wintermeyer et al. 2016), multiple enzymes and tissues can participate in this process and may compensate in the in vivo situation (Bouillon and Bikle 2019; Zhu et al. 2013). This may explain why human datasets do not consistently show parallel changes in both transcripts. Additionally, steatosis induces alterations in lipid metabolism, mitochondrial function, and xenobiotic processing, with notable remodeling of CYP enzymes and transporter proteins (Athinarayanan et al. 2021; Badmus et al. 2022; Naik et al. 2013; Rao et al. 2023). These adaptive responses can indirectly influence vitamin D hydroxylation. Furthermore, vitamin D is a fat-soluble vitamin. Hence, hepatic lipid accumulation and adipose tissue can modify its storage, transport, and release, further reducing its systemic availability (Aggeletopoulou et al. 2024; Nimitphong et al. 2020). Our platform effectively captures these hepatic adaptations under lipotoxic stress, and its human-based, multicellular design enables the study of these interactions without the species-specific confounders that often challenge translation from rodent models to humans (Babai et al. 2021; Tutty et al. 2022).

Although renal enzymes CYP27B1 and CYP24A1 critically regulate systemic 1,25(OH)D_3_ activation and degradation (Meyer and Pike 2020; Wintermeyer et al. 2016), our model focuses on hepatic 25-hydroxylation. Emerging evidence suggests that bone itself expresses CYP27B1, enabling the local conversion of 25(OH)D_3_ to the active form 1,25(OH)D_3_ in an autocrine manner, which promotes osteoblast differentiation and mineralization (Geng et al. 2013; van Driel and van Leeuwen 2023). Therefore, reduced hepatic 25-hydroxylation in our MASLD model may restrict the substrate required for bone-local activation (Geng et al. 2010; Lou et al. 2017). This offers a mechanistic explanation for impaired bone mineralization in MASLD. Future iterations incorporating renal components or measuring CYP24A1 and 24,25(OH)D_3_ levels could further clarify the systemic regulation of vitamin D availability to bone.

In our study, MASLD conditions resulted in a decrease in mineral density in the bone scaffolds, while the stiffness of the scaffolds remained unchanged. Additionally, only minor changes were observed in markers of osteoclastic activity. This pattern suggests an early mineral accrual deficit rather than established structural failure (Minisola et al. 2020; Wintermeyer et al. 2016). A similar phenotype has been reported in MASLD animal models, where male mice exposed to a Western diet for 16 weeks exhibited trabecular bone loss without compromising mechanical integrity. In contrast, prolonged exposure (48 weeks) resulted in cortical bone deterioration and reduced stiffness, indicating progressive skeletal weakening. This time-dependent in vivo outcomes reinforce the idea that MASLD initially disrupts mineral accumulation before structural failure becomes apparent (Goldscheitter et al. 2025).

This phenotype also aligns with vitamin D insufficiency, where osteoblast maturation and hydroxyapatite deposition are impaired before mechanical weakening becomes evident (van Driel and van Leeuwen 2023). Suboptimal 25(OH)D_3_ can therefore reduce mineral content even if some early osteogenic markers appear unchanged at intermediate time points. Moreover, vitamin D also modulates osteoblast-osteoclast homeostasis via regulatory signals such as RANKL/OPG signaling (Monti et al. 2024), offering an additional route through which hepatic damage may affect bone remodeling. Although we did not measure these ligands directly, the mineral phenotype observed is compatible with a state in which mineralization lags behind matrix formation. Furthermore, mitochondrial activity was significantly higher in MASLD-exposed bone scaffolds despite similar DNA content. This suggests increased cellular stress rather than improved mitochondrial functions. MASLD-related metabolic dysregulation is known to increase reactive oxygen species (ROS), which can lead to mitochondrial dysfunction and oxidative stress (Li et al. 2025; Radosavljevic et al. 2024). As a compensatory mechanism, cells may upregulate mitochondrial activity to maintain energy production and matrix synthesis under suboptimal conditions. However, this response may also perpetuate ROS generation, contributing to a cycle of oxidative damage and impaired bone remodeling (Aspera-Werz et al. 2018; Guo et al. 2022).

Several limitations of the present model should be acknowledged. Although the liver spheroid composed of HepaRG, LX-2, and HUVEC cells together with the bone scaffold containing SCP-1 and THP-1 cells captures selected aspects of the liver-bone axis, it remains a simplified system and does not fully reproduce MASLD-associated hepatic osteodystrophy in vivo. FFAs treatment induces steatotic and lipotoxic changes, but it does not encompass the full spectrum of endocrine, inflammatory, metabolic, and gut-derived signals involved in disease progression. In particular, the model lacks Kupffer cells and therefore does not fully recapitulate the hepatic immune microenvironment. It also lacks a renal component, precluding analysis of the complete systemic vitamin D axis. In addition, scaffold-based bone constructs do not reproduce the full native bone niche, including vascularization, osteocytes, and mechanical loading. Finally, the use of immortalized cell lines improves reproducibility but limits donor-specific variability and physiological heterogeneity.

An important aspect of the bone compartment also to consider is the use of THP-1 cells. THP-1 cells are widely used as a human monocyte/macrophage model and can differentiate into inflammatory macrophage phenotypes under appropriate stimulation. In our system, THP-1 cells were placed in a bone-specific microenvironment and primed with PMA, along with cues from the mineralized scaffold and SCP-1 cells, conditions intended to favor osteoclast-like differentiation (Li et al. 2017). It is also important to note that differentiation toward macrophage-like states is an inherent feature of osteoclast precursors, as osteoclasts themselves originate from the monocyte/macrophage lineage and can be considered specialized bone-resident macrophages. Therefore, some degree of macrophage-associated phenotype is expected regardless of the precursor used. Thus, although inflammatory liver-bone crosstalk may contribute to the overall phenotype, THP-1 cells in this model should primarily be interpreted as osteoclast precursors supporting the analysis of bone remodeling.

Compared with organ-on-chip platforms, our model represents a static 3D co-culture system rather than a microfluidically perfused one. Organ-on-chip systems can provide dynamic flow, shear stress, more physiologic nutrient and oxygen gradients, and improved inter-compartmental signaling, which may further influence hepatocyte function, endothelial behavior, and bone remodeling responses. At the same time, those platforms are technically more complex and less straightforward to scale for routine mechanistic studies. Our static model, therefore, prioritizes robustness, accessibility, and experimental throughput, whereas future integration of microfluidic perfusion could further enhance physiologic relevance and enable more refined analysis of liver-bone communication.

This study primarily aimed to establish a robust, mechanism-ready platform suitable for investigating the early stages of MASLD at the liver-bone axis and for evaluating targeted interventions. This human multicellular system overcomes several limitations common to animal studies and 2D cultures (Punt et al. 2017; Ruoss et al. 2020). HepaRG-based liver spheroids maintain metabolic competence and reproducibility, incorporating stellate and endothelial cells that shape the hepatic environment (Zahmatkesh et al. 2022). Weekly transfer of bone scaffolds onto freshly induced spheroids minimizes direct FFAs exposure to the bone compartment, increasing confidence that skeletal effects arise from liver‑derived soluble mediators. Excluding Kupffer cells means we do not fully simulate the inflammatory cascade typical of metabolic-associated steatohepatitis (MASH) (Tacke et al. 2023). However, this also has the benefit of allowing us to focus on hepatocellular mechanisms inherent to MASLD without immune amplification. In other words, the design choice that limits progression modeling actually improves our understanding of the hepatic enzymatic step involved in vitamin D metabolism.

In summary, our established in vitro platform successfully demonstrates that impaired hepatic vitamin D hydroxylation represents a central pathogenic mechanism in MASLD, with downstream implications for bone health. Clinically, these findings suggest that therapeutic strategies targeting the restoration of hepatic CYP activity or bypassing defective hydroxylation, may not only improve vitamin D status but also attenuate liver disease progression and consequently reduce fracture risk in patients with MASLD.

### Data availability

The original contributions presented in the study are included in the article. Further inquiries can be directed to the corresponding author. The Raw RNA sequencing data were derived from NCBI (accession number PRJEB58091) uploaded by the European Bioinformatics Institute.

## Supplementary Information

Below is the link to the electronic supplementary material.

Supplementary Material 1

## Abbreviations

25(OH)D_3_: 25-hydroxyvitamin D
ABCC1: ATP-binding cassette subfamily C member 1
AP: Alkaline phosphatase
COL1A1: Collagen type I alpha 1 chain
COL6A3: Collagen type VI alpha 3 chain
CPT1A: Carnitine palmitoyltransferase 1 A
CT: Computed tomography
CYP: Cytochrome P450
DHCR7: 7-dehydrocholesterol reductase
ECM: Extracellular matrix
FABP3: Fatty acid binding protein 3
FADS1: Fatty acid desaturase 1
FASN: Fatty acid synthase
FFAs: Free fatty acids
GSTA4: Glutathione S-transferase alpha 4
HMGCS2: 3-hydroxy-3-methylglutaryl-CoA synthase 2
IFN-γ: Interferon-γ
IL-1β: Interleukin 1 beta
IL-6: Interleukin 6
LDH: lactate dehydrogenase
LIPA: Lipase A, lysosomal acid type
MASLD: Metabolic dysfunction-associated steatotic liver disease
NAFLD: Non-alcoholic fatty liver disease
PC: Pyruvate carboxylase
PHH: Primary human hepatocytes
PINP: Procollagen type I N-terminal propeptide
RNA-seq: RNA sequencing
SLC16A7: Solute carrier family 16 member 7
TIMP1: Tissue inhibitor of metalloproteinases 1
TNF-α: Tumor necrosis factorα
TRAP: Tartrate-resistant acid phosphatase
VDR: Vitamin D receptor
