# Supplementary material for: Free Fatty acid-induced disruption of hepatic vitamin D metabolism impairs bone homeostasis in an in vitro 3D human liver–bone model
Source: Arch Toxicol. 2026 May 13;100(9):4151–69. doi: 10.1007/s00204-026-04431-x (PMC13454016; doi:10.1007/s00204-026-04431-x)
Supplement: Supplementary file 1 [file 204_2026_4431_MOESM1_ESM.docx]

**Free Fatty Acid-Induced Disruption of Hepatic Vitamin D Metabolism Impairs Bone Homeostasis in an *in Vitro* 3D Human Liver–Bone Model**

Mohammad Majd Hammour^1^, Lisa Herzberger^1,2^, Yuxuan Xin^1^, Guanqiao Chen^1^, Melike Tombaz^1^, Sabrina Ehnert^1^, Fabian Springer^3,4^, Georg Damm^5^, Massoud Vosough^6,7^, Jan G. Hengstler^8^, Ursula Müller-Vieira^2^, Andreas K. Nüssler^1,#^, and Romina H. Aspera-Werz^1,#,*^

^1^ Department of Traumatology, Siegfried Weller Institute, BG Clinic Tübingen, Eberhard Karls University, 72076 Tübingen, Germany

^2^ Boehringer Ingelheim Pharma GmbH & Co. KG, NCE, Biberach an der Riss, Germany

^3^ Department of Diagnostic and Interventional Radiology, University Hospital Tübingen, Hoppe-Seyler-Str. 3, 72076 Tübingen, Germany

^4^ Department of Radiology, BG-Klinik Tübingen, Eberhard Karls University, 72076 Tübingen, Germany

^5^ Department of Hepatobiliary Surgery and Visceral Transplantation, Clinic for Visceral, Transplant, Thoracic and Vascular Surgery, Leipzig University Medical Center, 04103 Leipzig, Germany

^6^ Department of Regenerative Medicine, Cell Science Research Center, Royan Institute for Stem Cell Biology and Technology, ACECR, Tehran, Iran

^7^ Experimental Cancer Medicine, Institution for Laboratory Medicine, Karolinska Institutet, Stockholm, Sweden

^8^ Leibniz Research Center for Working Environment and Human Factors (IfADo), 44139 Dortmund, Germany

**^#^**Equal contribution

*****Corresponding author: [Romina.Aspera-Werz@med.uni-tuebingen.de](mailto:andreas.nuessler@gmail.com)

Supplementary Figure 1. **Reversibility of FFA-induced steatosis in liver spheroids after fatty-acid withdrawal**.

Differentiated liver spheroids were exposed to 600 µM FFAs (2:1 oleic:palmitic acid) for 7 days to induce lipid accumulation and then switched to vehicle control medium (FFA withdrawal). The box plots show quantification of BODIPY fluorescence intensity normalized to nuclear staining (Hoechst). Data are shown as Tukey boxplots (N = 3; n = 2). Statistical significance was assessed using two-way ANOVA followed by Tukey's multiple comparisons test; **p < 0.01, ***p < 0.001.
